# BMP4 acts as a dorsal telencephalic morphogen in a mouse embryonic stem cell culture system

**DOI:** 10.1242/bio.012021

**Published:** 2016-11-04

**Authors:** Momoko Watanabe, Ernest S. Fung, Felicia B. Chan, Jessica S. Wong, Margaret Coutts, Edwin S. Monuki

**Affiliations:** 1 Department of Developmental and Cell Biology, School of Biological Sciences, University of California Irvine, Irvine, CA 92697-2300, USA; 2 Department of Pathology and Laboratory Medicine, School of Medicine, University of California Irvine, Irvine, CA 92697-4800, USA; 3 Sue and Bill Gross Stem Cell Research Center, University of California Irvine, Irvine, CA 92697-1705, USA

**Keywords:** Cortical hem, Choroid plexus epithelial cell, ES cell

## Abstract

The concept of a morphogen – a molecule that specifies two or more cell fates in a concentration-dependent manner – is paradigmatic in developmental biology. Much remains unknown, however, about the existence of morphogens in the developing vertebrate central nervous system (CNS), including the mouse dorsal telencephalic midline (DTM). Bone morphogenetic proteins (BMPs) are candidate DTM morphogens, and our previous work demonstrated BMP4 sufficiency to induce one DTM cell fate – that of choroid plexus epithelial cells (CPECs) – in a mouse embryonic stem cell (mESC) culture system. Here we used BMP4 in a modified mESC culture system to derive a second DTM fate, the cortical hem (CH). CH and CPEC markers were induced by BMP4 in a concentration-dependent manner consistent with *in vivo* development. BMP4 concentrations that led to CH fate also promoted markers for Cajal–Retzius neurons, which are known CH derivatives. Interestingly, single BMP4 administrations also sufficed for appropriate temporal regulation of CH, CPEC, and cortical genes, with initially broad and overlapping dose-response profiles that sharpened over time. BMP4 concentrations that yielded CH- or CPEC-enriched populations also had different steady-state levels of phospho-SMAD1/5/8, suggesting that differences in BMP signaling intensity underlie DTM fate choice. Surprisingly, inactivation of the cortical selector gene *Lhx2* did not affect DTM expression levels, dose-response profiles, or timing in response to BMP4, although neural progenitor genes were downregulated. These data indicate that BMP4 can act as a classic morphogen to orchestrate both spatial and temporal aspects of DTM fate acquisition, and can do so in the absence of *Lhx2*.

## Abstract (precis)

**Summary:** Using mouse embryonic stem cell cultures, we show BMP4 sufficiency to specify two cell fates and appropriate temporal patterning of the dorsal telencephalon, i.e. evidence for BMP4 as a dorsal telencephalic morphogen.

## INTRODUCTION

By definition, a morphogen is an instructive molecule that can specify two or more cell fates in a concentration-dependent manner (Ashe and Briscoe, 2006). In metazoans, morphogens often share other features including secretion from localized signaling centers, resulting in concentration gradients within target tissues, and the ability to act directly on cells at both short- and long-ranges (Grove and Monuki, 2013; Kicheva and Gonzalez-Gaitan, 2008; Tabata and Takei, 2004). Many such morphogens are now well known in invertebrate systems (Kicheva et al., 2007; Porcher and Dostatni, 2010). In vertebrate CNS model systems, classic morphogens are also thought to exist, including Sonic hedgehog (SHH) in the spinal cord, retinoic acid (RA) in the hindbrain, and fibroblast growth factors (FGFs) in the rostral-most telencephalon (Dessaud et al., 2007; Ericson et al., 1997; Schilling et al., 2012; Stamataki et al., 2005; Toyoda et al., 2010). These examples have relied largely on *in vivo* models, in which potentially overlapping, redundant, or interacting positional systems remain intact and are challenging to eliminate experimentally. *In vitro* systems, which allow for homogenization of positional information and enable formal testing for morphogen activity, have been more difficult to come by.

In this study we focus on the murine dorsal telencephalon, which consists of the dorsal telencephalic midline (DTM) and bilateral cerebral cortex. The DTM contains two distinctive bilateral structures that are derived from neuroepithelium: the choroid plexus (ChP) and the cortical hem (CH), which are separated at the immediate midline by the choroid plaque. The ChP is a distinctive papillary tissue with ChP epithelial cells (CPECs) that produce the cerebrospinal fluid (CSF) and form the blood-CSF barrier. The CH is a transient junctional tissue between the ChP and cerebral cortex, which functions as a hippocampal organizer (Mangale et al., 2008) and source of Cajal-Retzius (CR) neurons (Meyer, 2010; Molyneaux et al., 2007; Monuki et al., 2001).

Previous studies have implicated BMPs as potential morphogens for these DTM fates. BMPs are produced at high levels in the dorsal telencephalic roof plate of the early neural tube (Furuta et al., 1997), and an intact roof plate is required for the continuous BMP signaling gradient that characterizes the normal dorsal telencephalon (Cheng et al., 2006). This endogenous gradient is also evident from position- and orientation-dependent effects induced by BMP4-soaked beads in explants (Hu et al., 2008). Moreover, an intact roof plate and BMP receptors are required for CH and CPEC specification in mice (Cheng et al., 2006; Currle et al., 2005; Fernandes et al., 2007; Hébert et al., 2002). These studies demonstrate that the dorsal telencephalon possesses a BMP signaling gradient, and that BMP sources and receptors are required for DTM fates.

However, evidence for BMP sufficiency to specify DTM fates, the *sine qua non* for a morphogen, is more limited. When applied to dissociated cortical progenitors, exogenous BMP4 can modestly upregulate CPEC genes in a concentration-dependent fashion, but does not cause CPEC respecification (Hu et al., 2008). BMP4 also suffices to partially rescue CPEC fate in roof plate-ablated explants (Cheng et al., 2006) and to ectopically induce CPEC fate towards the rostral midline in wild-type explants (Srinivasan et al., 2014). In addition, BMP4 is sufficient to specify CPEC fate from mESC-derived neuroepithelial cells (NECs) (Watanabe et al., 2012). These mESC-derived CPECs have properties that are indistinguishable from primary CPECs, and, consistent with *in vivo* and experimental studies (Thomas and Dziadek, 1993), CPEC competency is restricted to preneurogenic NECs rather than later-stage neurogenic radial glia. Likewise, the critical period for CH fate determination [embryonic day (E)8.5-10.5] maps to the preneurogenic NEC period (Mangale et al., 2008). The mESC culture system therefore provides an ideal *in vitro* model system to examine BMP4 sufficiency to induce CH in addition to CPEC fate. If consistent with *in vivo* development, CH cells should be specified at lower BMP4 concentrations than those required for CPEC fate.

In this paper, we utilize a modified mESC culture system to demonstrate BMP4 concentration dependence for CH and CPEC fates *in vitro*, consistent with *in vivo* development. In the modified system, aggregated mESC-derived NECs are dissociated into monolayers to allow for uniform exposure to exogenous BMP4. Importantly, single administrations of BMP4 also induce temporal patterns in CH and CPEC gene expression that mimic *in vivo* development, including sharpening of dose-response profiles over time. Thus, BMP4 alone can specify not only multiple DTM fates, but also appropriate temporal patterning of DTM gene expression.

## RESULTS

### BMP4 concentration-dependent induction of CH and CPEC markers in a modified mESC culture system

**Fig. 1.**
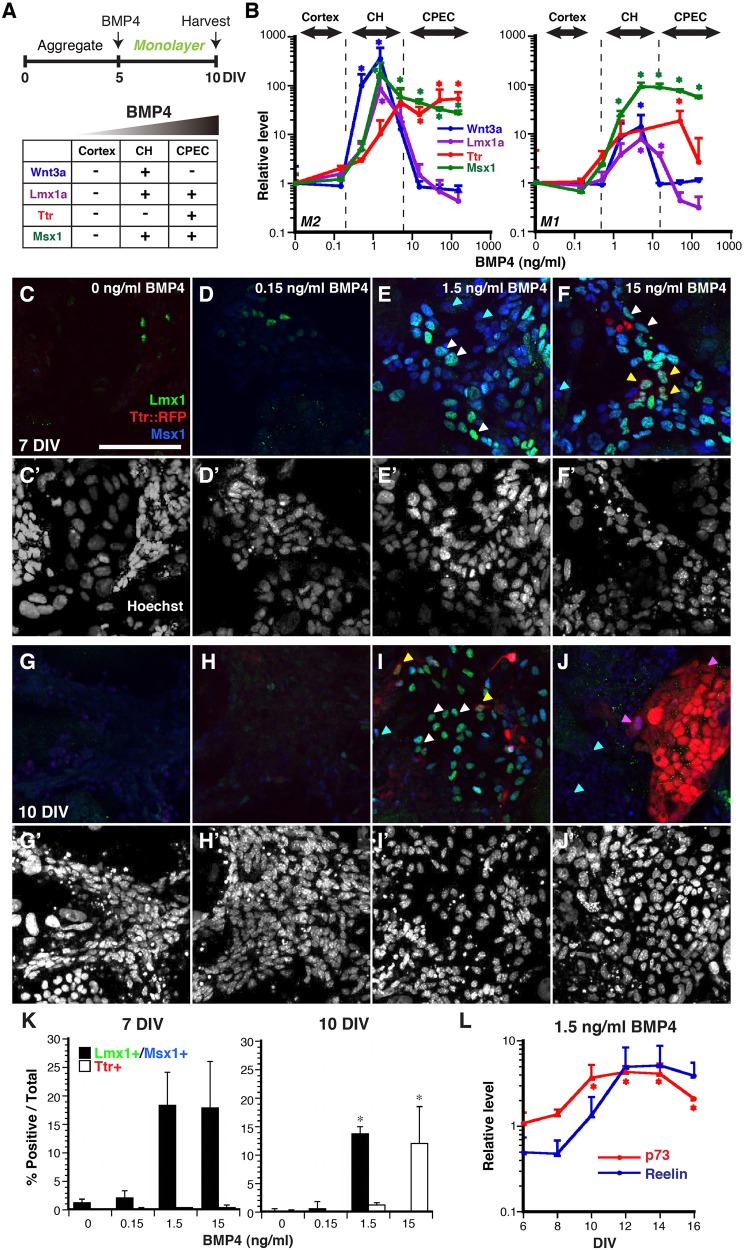
**BMP4 dose-dependency to drive CH and CPEC fates.** (A) Schematic of the monolayer (M) system to induce CH and CPEC fates from mESCs and the chart for DTM expression profile. (B) RT-qPCR of dissociated 5-day M2 and M1 aggregates with a single application of BMP4 at 10 DIV. Three BMP4-dose ranges are found to preferentially regulate cortex, CH, and CPECs markers. (C-J) Immunocytochemical analysis of dissociated 5-day M2 aggregates treated with BMP4 for another 2 DIV (C-F) or 5 DIV (G-J). Lmx1 (green) and Msx1 (blue) are initially highly upregulated with 1.5 ng/ml and 15 ng/ml BMP4 at 7 DIV, and restricted to 1.5 ng/ml BMP4 culture at 10 DIV. Ttr::RFP takes time to upregulate mainly in 15 ng/ml BMP4 culture and few in 1.5 ng/ml BMP4 culture. Corresponding fields for Hoechst staining (white) are shown in C′-J′ for cell density comparison. Blue arrowheads: Msx1 single positive; white arrowheads: Msx1/Lmx1 double positive; yellow arrowheads: Msx1/Lmx1/Ttr triple positive; pink arrowheads: Ttr single or Ttr/Msx1 double positive. Scale bar: 100 μm. (K) Quantification of DTM markers represented in C-J. Immuno-positive cells/total cells (Hoechst). Because C-J are magnified selected fields, lower magnification pictures are provided in Fig. S2. (L) Enriched Cajal-Retzius (CR) markers from CH-abundant cultures. M2 dissociated 5-day aggregates treated with 1.5 ng/ml (RT-qPCR, normalized to no BMP4 control at each time point) demonstrated increased levels of CR markers, *p73* and *Reelin*, in a temporally sequential manner. For K and L, data are presented as mean±s.e.m.; *P<0.05 compared to no BMP culture.

We first used our existing mESC aggregate cultures (Watanabe et al., 2012) to look for CH marker induction across an extensive range of BMP4 concentrations (0.1-512 ng/ml; Fig. S1B, M1 mESC line). In our previous study (Watanabe et al., 2012), we used five different CPEC markers that displayed similar BMP4 dose-response profiles (*Ttr*, *Msx1*, *Aqp1*, *Cldn1*, and *Lmx1a*) and confirmed CPEC identity by ultrastructural analyses and functional assays. Since Ttr expression is particularly high and exclusive to CPECs, Ttr (or TTR::RFP) was used to follow CPEC differentiation here. In contrast to CPECs, CH cells should be negative for *Ttr* while being positive for *Msx1*, *Lmx1a*, and *Wnt3a* (Fig. 1A); however, we did not find a BMP4 concentration that induced a CH-like profile in the aggregate system, as all three genes displayed similar dose-response profiles (Fig. S1B). One potential explanation for this is that cells in aggregates are exposed to widely varying extracellular BMP4 concentrations depending on their radial positions, a natural limitation of aggregates in general.

We therefore developed a monolayer culture system to expose cells to more uniform BMP4 concentration. In this system, after 5 days *in vitro* (DIV) of neural induction, aggregates were dissociated into single cells and plated onto an adherent surface, then exposed to exogenous BMP4 at varying concentrations (0.15-150 ng/ml) for an additional 5 DIV (Fig. 1A). Using two different mESC lines, we found that, unlike the aggregate-only system, moderate BMP4 concentrations (0.5-5 ng/ml for M2 and 1.5-5 ng/ml for M1 cells) induced a CH-like profile, with relatively high *Wnt3a*, *Lmx1a*, and *Msx1* compared to *Ttr*; this was particularly clear for the M2 line (Fig. 1B). In addition, at the highest BMP4 concentrations, CH-specific *Wnt3a* was strongly suppressed, while the Ttr was upregulated and high *Msx1* was maintained, consistent with a CPEC profile (Fig. 1B). In the low BMP4 range (<0.15 ng/ml for M2 and <0.5 ng/ml for M1), DTM markers were not detectably induced; therefore, this range may correspond to the lower BMP signaling levels seen in the developing cortex (Fig. S1A) (Cheng et al., 2006). Thus, both CH and CPEC gene expression profiles could be induced in a BMP4 concentration-dependent manner in the same monolayer cultures.

To confirm CH identity at the single cell level, immunocytochemical analyses were performed. Based on the markers used, CH cells should express Lmx1a and Msx1, but not TTR::RFP. We found that, in the low BMP4 range (0 or 0.15 ng/ml), very few cells were positive for Msx1, Lmx1a, or TTR::RFP after 7 DIV (5 DIV aggregate plus 2 DIV monolayer) or 10 DIV (5 DIV aggregate plus 5 DIV monolayer; Fig. 1C-D,G-H), similar to the RT-qPCR studies (Fig. 1B). At moderate BMP4 concentration (1.5 ng/ml), Lmx1a/Msx1 double-positive cells were observed at 7 DIV (18.25±5.85%) and 10 DIV (13.83±1.23%), but few TTR::RFP-expressing cells were detected (Fig. 1E,I,K), which corresponds well to the CH gene expression profile (Fig. 1A). In cultures with the highest BMP4 concentration (15 ng/ml), Lmx1a/Msx1 double-positive cells were detected at 7 DIV (17.83±8.14%), but not at 10 DIV, while TTR::RFP expression displayed the converse pattern, low at 7 DIV (0.42±0.42%) but prominent at 10 DIV (12.00±6.46%) (Fig. 1F,J,K), with the TTR::RFP-expressing cells displaying a more flattened appearance typical of CPECs and other epithelial cells in culture (Fig. S2I-L). CPEC yield was approximately three to four times higher in this modified monolayer culture system compared to the aggregate system. Thus, the 10 DIV cultures most clearly distinguished the CH cells and CPECs, and further supported the BMP4 concentration dependence (1.5 ng/ml BMP4 for CH, 15 ng/ml BMP4 or higher for CPEC) seen by population RT-qPCR analysis (Fig. 1B). Interestingly, CPEC induction at 15 ng/ml BMP4 was preceded at 7 DIV by a more CH-like molecular phenotype; this raises the possibility that CPEC development involves an intermediate CH-like stage, a point that is further addressed below.

### Co-enrichment for CH and Cajal-Retzius (CR) markers at moderate BMP4 concentration

In addition to being a hippocampal organizer (Mangale et al., 2008), the CH also generates CR neurons (Molyneaux et al., 2007). To further evaluate CH induction in our system we examined CR neuron markers. While Reelin-expressing CR cells have multiple origins in addition to the CH (Meyer, 2010; Molyneaux et al., 2007), p73 expression is restricted to CH-derived CR neurons and precedes Reelin expression (Meyer et al., 2002).

We therefore examined *p73* and *Reelin* expression, and their temporal expression patterns over a longer time period (6-16 DIV). At 1.5 ng/ml BMP4 – the concentration yielding maximal CH marker expression (Fig. 1) – *p73* and *Reelin* inductions were detected by 10 DIV (Fig. 1L). The *p73* expression plateau was reached by 10 DIV, whereas the plateau for *Reelin* expression was not reached until 12 DIV (Fig. 1L). In contrast, at high BMP4 concentration (15 ng/ml), neither *p73* nor *Reelin* expression increased above baseline levels during the 6-16 DIV period (Fig. S3A). Thus, the BMP4 dose-response profile for CR neuron markers matched that of the CH rather than CPECs. Moreover, the observed temporal profiles for *p73* and *Reelin* are consistent with those seen *in vivo* for CH-derived CR cells (Meyer et al., 2002).

### Temporal regulation of DTM markers in a BMP4 dose-dependent fashion

**Fig. 2.**
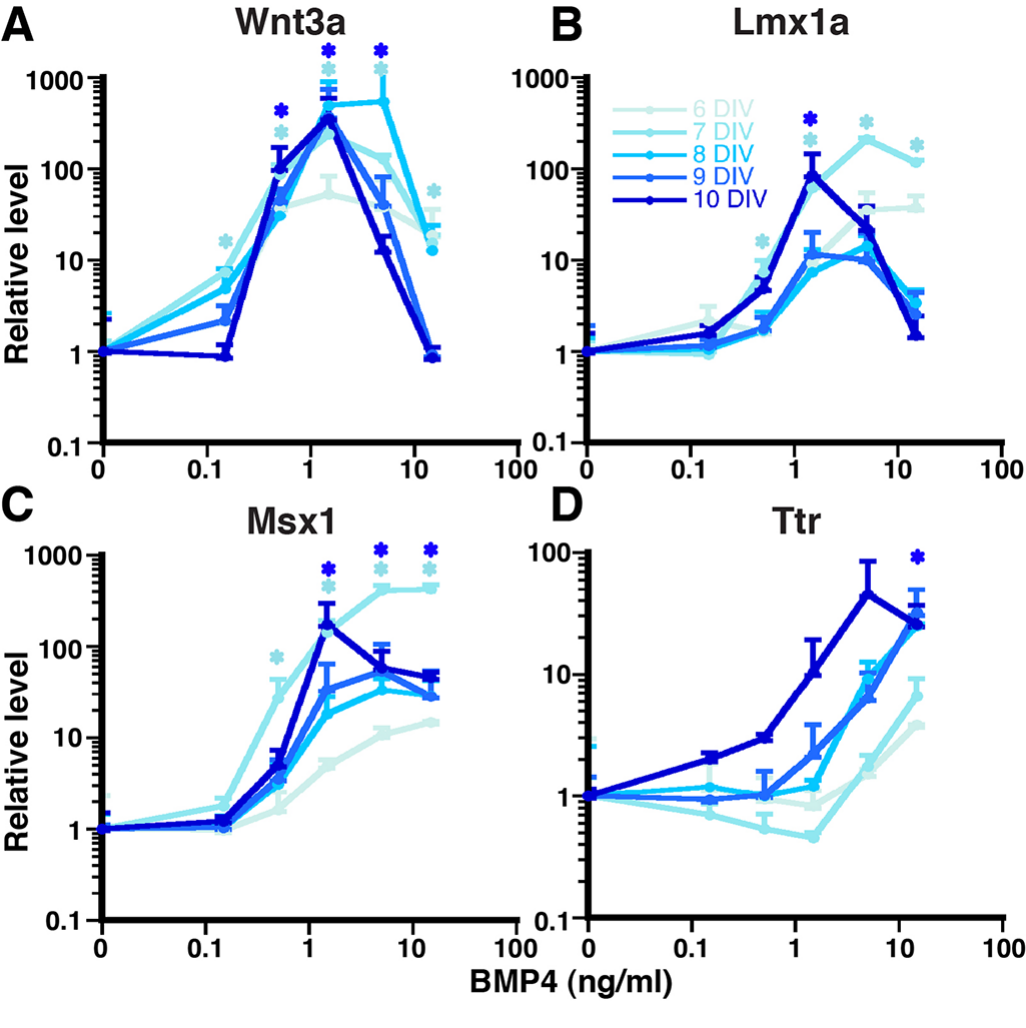
**Temporal regulation of DTM markers by BMP4.** (A-D) RT-qPCR of dissociated 5-day M2 aggregates treated with BMP4 (0.15-15 ng/ml) for another 1-5 DIV. (A,B) CH markers, *Wnt3a* and *Lmx1a*, are increased quickly at moderate-high BMP4 concentration (0.15-15 ng/ml). However, CH markers are maintained only at moderate BMP4 concentration (0.5-1.5 ng/ml for *Wnt3a*, 1.5-5 ng/ml for *Lmx1a*), while they are downregulated at high (>5 ng/ml) BMP4 concentration. (C) *Msx1* is a direct target of BMP signaling and activated at moderate-high BMP4 concentration (0.5-15 ng/ml) at early to late time points. (D) *Ttr* is a mature CPEC marker and activated only at high BMP4 concentration (>5 ng/ml) at later time points (after 8 DIV), consistent with *Ttr* being a mature late-onset CPEC marker *in vivo*. Similar results are obtained, using the M1 mESC line (Fig. S4). Data are presented as mean±s.e.m.; *P<0.05 compared to no BMP culture.

We then focused on other temporal aspects of DTM gene expression following a single application of BMP4, since in addition to determining spatial patterns, morphogens are thought to determine temporal aspects of patterning *in vivo* (Dessaud et al., 2007; Tozer et al., 2013). M2 5-day SFEBq aggregates were dissociated and plated with varying concentrations of BMP4 (0.15-15 ng/ml), with gene expression examined after 1-5 DIV (6-10 DIV total). RT-qPCR assays revealed that CH genes *Wnt3a* and *Lmx1a* were induced quickly at both moderate and high BMP4 concentrations (0.15-15 ng/ml) within 1-3 DIV. Over time, these genes were maintained at the moderate BMP4 concentrations, but suppressed at the high ones (Fig. 2A,B). These temporal- and concentration-dependent patterns coincide with those seen in the immunocytochemical analyses (Fig. 1K).

Different temporal patterns were observed for *Msx1* and CPEC-specific *Ttr*. *Msx1* was not only induced, but also maintained at moderate and high BMP4 (0.5-15 ng/ml) (Fig. 2C), consistent with *Msx1* being a positional determinant of high BMP signaling (Cornell and Ohlen, 2000; Ramos and Robert, 2005) and its maintenance in adult CPECs *in vivo* (Ramos et al., 2004), unlike *Lmx1a* (Zou et al., 2009). In contrast, *Ttr* levels were relatively low at early time points and increased over time with the highest levels occurring at the highest BMP4 concentrations tested (Fig. 2D), consistent with *Ttr* being a relatively late CPEC marker *in vivo* compared to *Msx1* and *Lmx1a* (Currle et al., 2005; Hu et al., 2008). Similar temporal- and concentration-dependent expression patterns for these DTM markers were observed using a different mESC line (Fig. S4). Interestingly, the concentration-response profiles also sharpened (i.e. the slopes between points steepened) with increasing time. Since this sharpening occurs in monolayer cultures (rather than intact tissues with localized signaling sources and other positional cues), it raises the possibility of cell-intrinsic ultra-sensitivity to BMP4 similar to that seen in primary cortical progenitors (Hu et al., 2008; Srinivasan et al., 2014). Taken together, these data indicate that a single BMP4 dose in the mESC-based system was sufficient for temporal DTM gene patterning and sharpening that recapitulates *in vivo* development.

### Differential steady-state levels of BMP signaling correlate with the two cell fates

BMP signaling is directly transduced into the tail phosphorylation of Smad1/5/8 (Massague et al., 2005), leading us to wonder about the levels and dynamics of Smad1/5/8 activation in our culture system. To examine the effects of moderate (CH-inducing; 1.5 ng/ml) and high (CPEC-inducing; 64 ng/ml) BMP4 concentrations on intracellular signaling, we measured phospho-Smad1/5/8 (pSmad) levels by western blot over time in 5-day SFEBq dissociated cells. At all time points examined, actin-normalized pSmad levels were higher in the cells treated with higher BMP4 (Fig. S3B). As in primary cortical progenitors (Hu et al., 2008), pSmad activation occurred rapidly in response to BMP4 (by 60 min, the earliest time point in these studies) and preceded the induction of DTM mRNAs (Fig. 2A,B). At later time points, pSmad levels remained elevated in cells treated with high BMP4 (Fig. S3B), although this could reflect excess BMP4 in the media rather a difference in cell biology per se (i.e. with high BMP4 concentrations in media intracellular BMP degradation could be limiting, whereas BMP4 availability could be the limiting factor at lower BMP4 concentrations). Regardless, the findings suggest that intracellular pSmad levels correlate positively with extracellular BMP4 concentration in this system.

### BMP4 regulates neural progenitor markers in a dose-dependent fashion

In addition to DTM fates, BMP signaling has been implicated in regulating cortical progenitors, and specifically, their expression of transcription factors (TFs). Within the E10.5-E12.5 cortical anlagen, BMP/pSmad signaling exhibits a high dorsal-low ventral (DV) gradient, the same DV polarity as the gradients for TFs Lhx2 and Emx2, while having the opposite polarity of the high ventral-low dorsal (VD)-graded TFs Pax6, Foxg1, and Ngn2 (Cheng et al., 2006). After reducing and flattening the BMP/pSmad gradient via roof plate ablation, the cortical TFs were differentially affected; the DV gradients (Lhx2 and Emx2) were also reduced and flattened, while the VD gradients (Pax6, Foxg1, and Ngn2) were relatively unaffected (Cheng et al., 2006). This raised the possibility that among the cortical TFs examined, BMP/pSmad signaling selectively upregulates Lhx2 and Emx2.

**Fig. 3.**
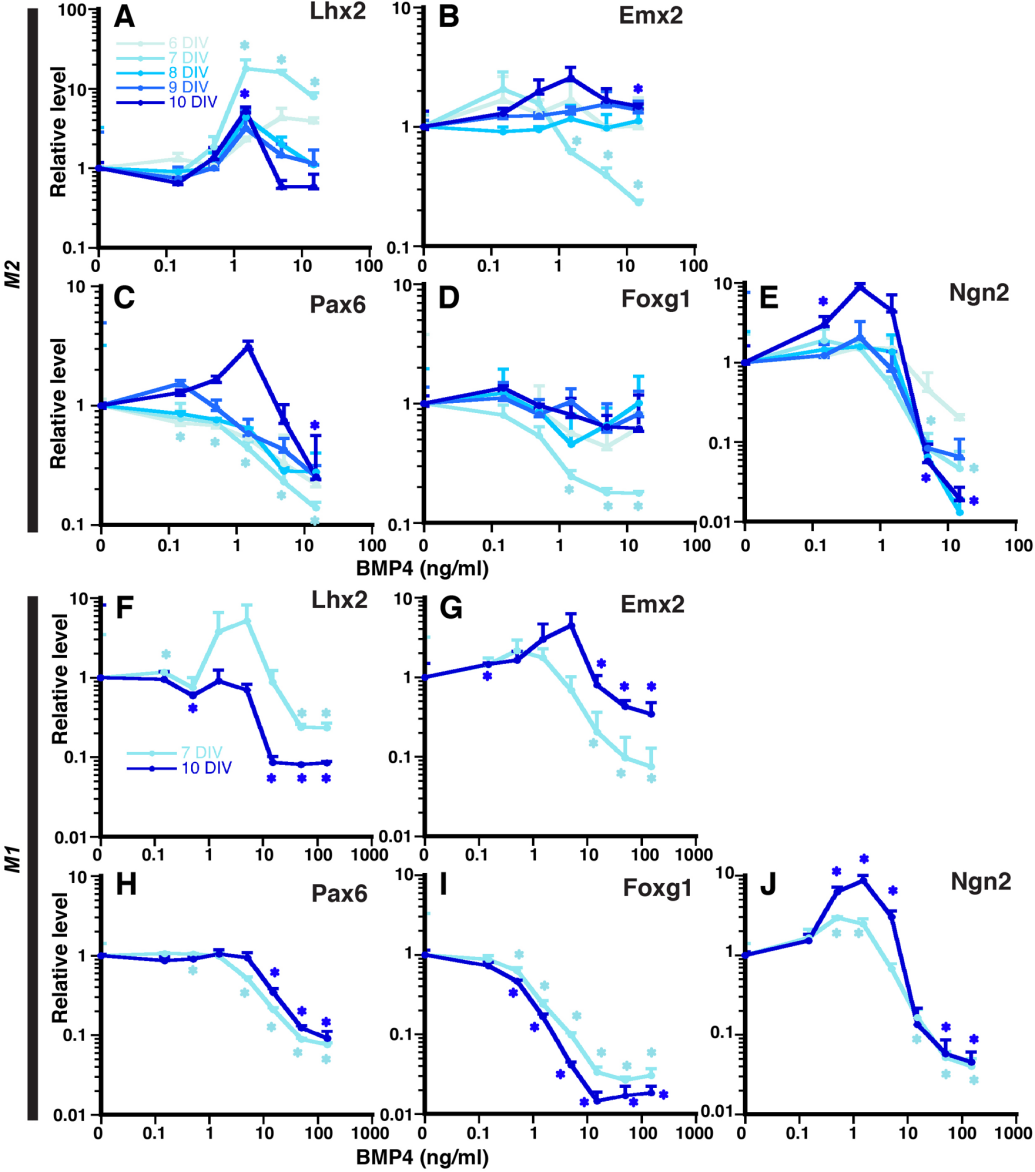
**BMP4 mediated-neural progenitor marker regulation in a dose-dependent fashion.** (A-J) 5-day SFEBq aggregates are dissociated and plated in monolayer with varying BMP4 (0.15-15 ng/ml for M2 and 0.15-150 ng/ml for M1) for another 1-5 DIV (6 to 10 DIV total). Usually, at higher BMP4 concentration, all neural progenitor expression is suppressed. Selectively, some neural progenitor markers are slightly upregulated in a temporal manner at lower concentration of BMP4. Data are presented as mean±s.e.m.; *P<0.05 compared to no BMP culture.

To address this potential selectivity, we examined the same DV- and VD-graded genes in our monolayer culture system using the M1 and M2 mESC lines. These analyses showed that the DV markers, *Lhx2* and *Emx2*, were upregulated at intermediate BMP concentrations, but interestingly, with different timing (Fig. 3A,B, best seen in Fig. 3F,G), *Lhx2* activation occurred earlier than *Emx2*, consistent with *Lhx2* being upstream of *Emx2* and other cortical transcription factors (Mangale et al., 2008). At these same intermediate BMP4 concentrations there were mixed VD gene patterns. *Pax6* was not consistently altered (Fig. 3C,H), *Foxg1* was slightly downregulated (Fig. 3D,I), and *Ngn2* was upregulated (Fig. 3E,J); generally high BMP4 concentrations downregulated all DV and VD markers. These results support the hypothesis that BMP signaling selectively upregulates DV markers, *Lhx2* and *Emx2*, at intermediate concentrations and can account for the *Lhx2* and *Emx2* findings in roof plate-ablated mutants (Cheng et al., 2006) as well as the *Lhx2* upregulation at a distance away from BMP4-soaked beads in cortical explants (Monuki et al., 2001). However, there were inconsistent effects on VD genes that cannot alone explain the VD gene expression phenotypes in these same mutants.

### Lhx2 inactivation does not facilitate BMP4-mediated DTM induction in culture

We then studied the influence of the *Lhx2* cortical selector gene on DTM induction by BMP4. *Lhx2* is expressed by cortical progenitor cells (hippocampal and neocortical), but not by CPECs or CH cells, and constitutive *Lhx2* absence results in excessive CH and CPEC fates (Monuki et al., 2001). This DTM fate suppression by *Lhx2* is also seen in genetic mosaics, with *Lhx2* null cells in the hippocampal primordium adopting CH, but not CPEC, fate in cell-autonomous fashion (Mangale et al., 2008). One possibility for the ectopic CH fate is increased BMP signaling intensity in *Lhx2* null cells, but pSmad and BRE-gal studies in *Lhx2* mosaic embryos argue against this (Doan et al., 2012). Another possibility is that *Lhx2* null cells are intrinsically biased to generate DTM fates even at lower BMP concentration and signaling intensity.

**Fig. 4.**
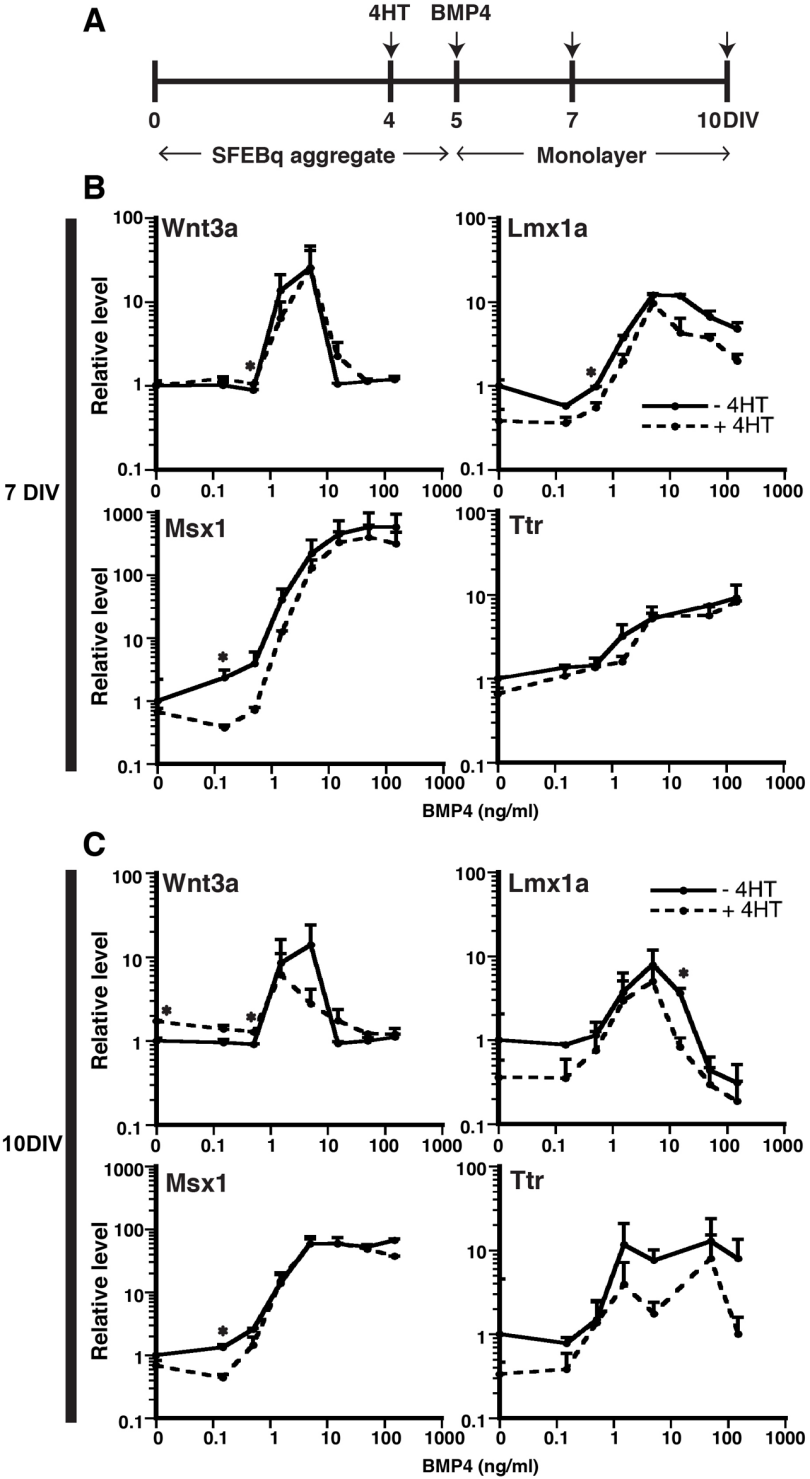
**No facilitation of BMP4-mediated DTM upregulation by *Lhx2* inactivation.** (A) Experimental design. All SFEBq aggregates are treated with (dotted lines) or without (solid lines) 4HT (1 μM) at 4 DIV. After 24 h, *Lhx2* is inactivated to about 95% (Fig. S5). 5-day SFEBq aggregates are then dissociated to single cells in monolayer with fresh media containing varying BMP4 concentrations (0.15-150 ng/ml) for another 2 DIV (B) or 5 DIV (C) for RT-qPCR analyses. All data points are normalized to monolayer cultures without BMP4 and 4HT. No significant gene expression change was detected, indicating that *Lhx2* absence does not facilitate BMP4-mediated DTM induction. Data are presented as mean±s.e.m.; *P<0.05 compared to no BMP culture.

We explored this possibility here by performing BMP4 dose-response experiments after *Lhx2* inactivation in cultured mESCs. Using M1 cells (*R26^CreER/+^Lhx2^cKO/cKO^*; Fig. S5A) (Watanabe et al., 2012), we developed a highly efficient and dose-dependent method for inactivating *Lhx2* using 4-hydroxytamoxifen (4HT), with maximal inactivation achieved at and above 1 μM 4HT (Fig. S5B). 4HT addition before 4 DIV adversely affected neuroepithelial induction (data not shown). We therefore applied 4HT at 4 DIV (Fig. 4A). At 5 DIV SFEBq aggregates were dissociated, plated as monolayers with varying amounts of BMP4 (0.15-150 ng/ml), then analyzed after 2 or 5 DIV (7 or 10 DIV total). In these cultures *Lhx2* was effectively inactivated at the genomic (Fig. S5C) and mRNA levels (96.47±2.92% reduction; Fig. S5D). Interestingly, the DTM gene profiles at both 7 and 10 DIV were quite similar between control and 4HT-treated cells (Fig. 4, solid and dashed lines, respectively) with no evidence of a left-shift or altered expression levels in the 4HT-treated cells (Fig. 4B,C). We performed similar studies with *Lhx2* inactivation at 5 DIV, and again saw no obvious effects on the levels, timing, or concentration-dependence of the DTM genes to BMP4 despite highly efficient *Lhx2* inactivation (data not shown). Thus, *Lhx2* inactivation did not influence BMP4-mediated DTM induction in this mESC culture system.

Since BMP4 can also regulate DTM genes in primary cortical progenitors (Hu et al., 2008), we examined BMP4 concentration-response profiles in primary cortical progenitors lacking *Lhx2* (*Emx1^Cre/+^;Lhx2^cKO/sKO^*; Mangale et al., 2008). In *Lhx2* null E11.5 progenitors, *Lhx2* mRNA levels were reduced by 81.76±9.05% compared to controls before plating (Fig. S5E). However, this *Lhx2* reduction did not further promote DTM gene expression at any BMP4 concentration (Fig. S5F). *Lhx2* inactivation therefore did not facilitate BMP4-mediated DTM induction in either the primary cortical progenitor or mESC culture system.

### Inactivation of Lhx2 downregulates other cortical progenitor markers

**Fig. 5.**
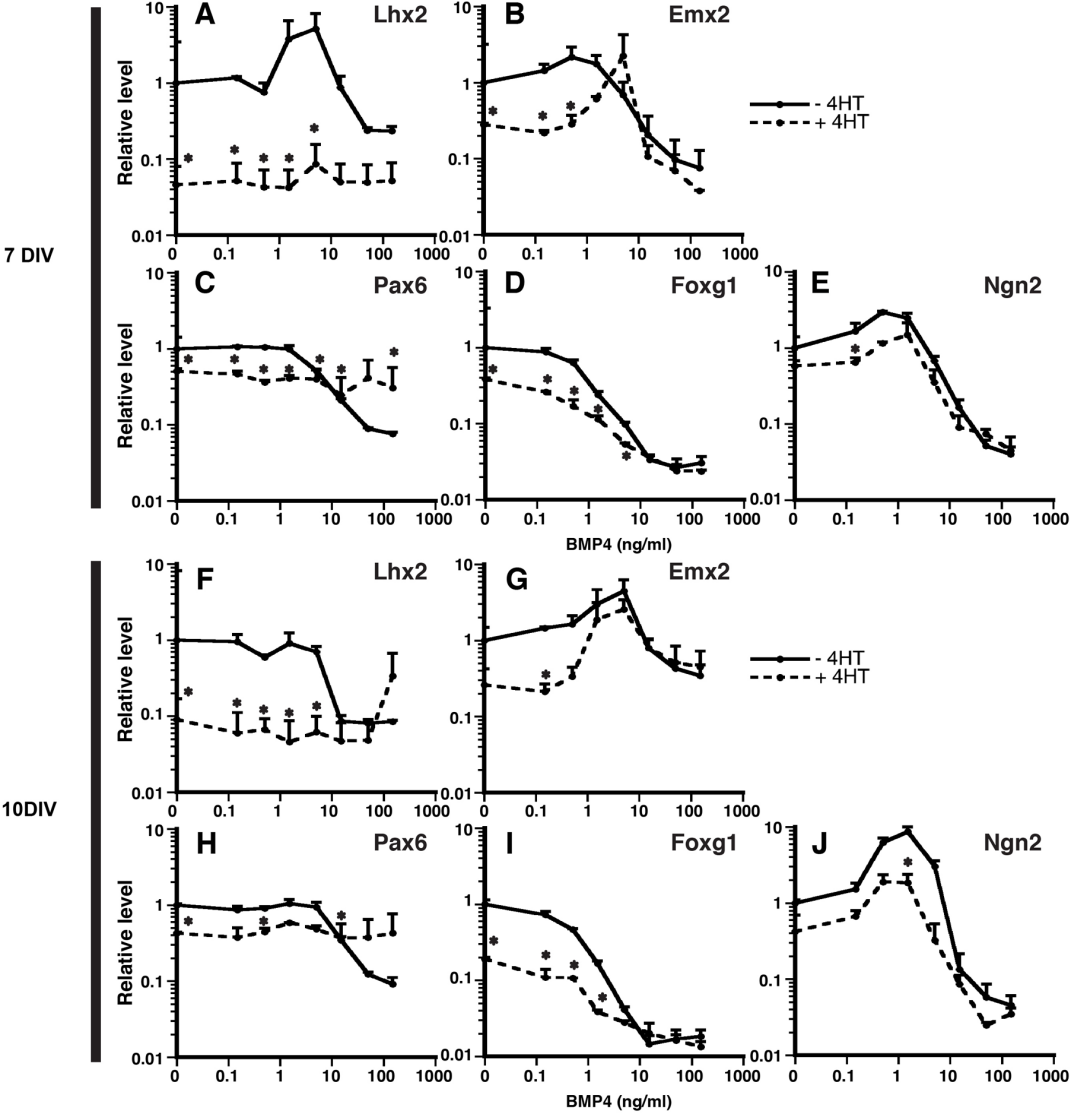
**Downregulation of other neural progenitor markers by inactivation of *Lhx2*.** 1 μM of 4HT or vehicle is applied to 4-day SFEBq aggregates and 5-day aggregates are dissociated with varying BMP4 concentrations (0.15-150 ng/ml). Gene expression is analyzed by RT-qPCR 2 DIV (A-E) or 5 DIV (F-J) later. At no-moderate BMP concentration (around 0-1.5 ng/ml), inactivation of *Lhx2* reduces all neural progenitor markers, consistent with *Lhx2* being an early hierarchy of telencephalic development. BMP4-mediated suppression of neural progenitor markers is not influenced by *Lhx2* inactivation except *Pax6* that is genetically linked with *Lhx2*. This indicates that *Lhx2* does not directly interact with BMP signaling. Data are presented as mean±sem; *P<0.05 compared to no 4HT culture.

As a cortical selector gene, *Lhx2* is at or near the top of the transcriptional hierarchy that specifies neuroepithelial cells with cortical identity (Mangale et al., 2008; Monuki et al., 2001). We therefore asked whether *Lhx2* inactivation (by 4HT addition at 4 DIV) influenced BMP4-mediated regulation of other cortical progenitor genes. These experiments revealed two consistent gene expression patterns by RT-qPCR analyses: first, at low to intermediate BMP4 concentrations (0-1.5 ng/ml), *Lhx2* inactivation caused reductions for all of the other genes (*Emx2* and VD genes) at both 7 and 10 DIV (Fig. 5A-J). This supports the view that *Lhx2* is upstream of these cortical progenitor genes. Second, at both 7 and 10 DIV, *Lhx2* inactivation had no effect on the suppression of *Emx2*, *Foxg1*, or *Ngn2* at high BMP4 concentrations, but interestingly, abrogated the suppression of *Pax6* (Fig. 5A-J). Thus, *Lhx2* was not required for BMP4-mediated suppression of *Emx2*, *Foxg1*, or *Ngn2*, but was required for that of *Pax6*. Together, these data show that *Lhx2* inactivation reduces cortical progenitor gene expression, but does not interfere with BMP4-mediated down-regulation of these genes, with the exception of *Pax6*.

## DISCUSSION

In this study two DTM fates, CH and CPEC, were induced from mESC-derived neuroepithelial progenitors, with a single BMP4 dose sufficing to recapitulate *in vivo* spatial patterning. In addition, single BMP4 doses sufficed to recapitulate the normal temporal patterning of DTM gene expression, as well as some aspects of selective cortical TF regulation seen *in vivo*. Taken together, the results from a reduced *in vitro* system, which minimizes confounds inherent in *in vivo* studies, provide evidence for BMP4 activity as a classical morphogen in the dorsal telencephalon. Interestingly, inactivation of *Lhx2*, which leads to excessive DTM fates *in vivo*, did not affect the levels, timing, or dose-response profiles of BMP4-mediated DTM marker induction *in vitro*; however, *Lhx2* inactivation downregulated cortical TF gene expression overall and abrogated BMP4-mediated suppression of *Pax6*. While consistent with *Lhx2* being a cortical selector gene (Mangale et al., 2008), these studies suggest that *Lhx2* neither mediates nor regulates the fundamental morphogenic activities of BMP4 during dorsal telencephalic development.

### The mouse dorsal telencephalon as a classic BMP morphogen gradient system

CH inducibility by BMP4 complements previous findings on the induction (Srinivasan et al., 2014; Watanabe et al., 2012) or rescue of CPECs (Cheng et al., 2006). Previous unsuccessful attempts to induce CH fate with BMP4 (Furuta et al., 1997; Hu et al., 2008; Monuki et al., 2001) may relate to the early and short competency period for CH fate *in vivo* (E8.5-E10.5 in mice; Mangale et al., 2008). Using dissociated neuroepithelial aggregates BMP4 also recapitulated the concentration (CH fate at lower BMP4 concentration than CPEC, Fig. 1B) and temporal profiles (CH fate before CPEC fate, Figs 1 and 2) expected for a DTM morphogen. Unfortunately, CH grafts to test for hippocampal organizer activity have been challenging (Tole and Grove, 2001), due in part to the early critical period (Mangale et al., 2008), and further grafting or co-culture advancements will be needed to further assess the derived CH cells. In addition, BMP4-mediated cell death and proliferation were not addressed here. CPECs and CH cells differentiate in domains of low proliferation and high death (Furuta et al., 1997; Currle et al., 2005), BMP4 can induce these fates (Furuta et al., 1997; Cheng et al., 2006; Watanabe et al., 2012), and BMP4 dose-response curves for these fates overlap (Mabie et al., 1999). Thus, proliferation and death effects are known and expected, and relevant BMP-mediated fates in our system, which are not accounted for in the normalized RT-qPCR data, reflect ‘per cell’ averages of viable populations.

Importantly, together with previous studies, our findings establish the mouse dorsal telencephalon as a classic morphogen gradient system (Grove and Monuki, 2013). In this system, BMP4 and other BMPs produced at the midline (Furuta et al., 1997; Monuki et al., 2001) lead to a continuous BMP/pSmad signaling gradient (Cheng et al., 2006) with a length scale of 270-290 μm at E10.5 (Srinivasan et al., 2014). This gradient also gives rise to graded and oriented responses around BMP4-soaked beads (Hu et al., 2008). BMP-producing cells (Cheng et al., 2006; Currle et al., 2005) and BMP receptors (Hébert et al., 2002) are also required for DTM fates. The ability of BMP4 to induce two DTM fates (CPECs and CH cells) represents a final piece of evidence for the classic model.

An additional point worth noting is the apparent CH-like intermediate state during CPEC differentiation, i.e. CH markers Wnt3a and Lmx1a were initially induced then extinguished at CPEC-inducing BMP4 concentrations (Figs 1 and 2). This concept is consistent with Wnt3a and Lmx1a expression *in vivo*, as well as *Wnt3a* genetic lineage studies (Chizhikov et al., 2010; Louvi et al., 2007). While potentially consistent with temporal integration of BMP signaling, BMP4 time-response data were not clear in this regard (data not shown). A CH-like intermediate state would also complicate previous CH-CPEC lineage analyses and questions about CPECs being hem derivatives. Regardless, a possible CH-like intermediate state will be important to account for in future CPEC studies and models.

### Selective BMP regulation of cortical patterning genes

Overall, our findings align well with previous BMP cortical gene studies. High BMP4 concentrations (i.e. the DTM regime) consistently suppressed the five cortical TF genes tested at both 7 and 10 DIV (Fig. 3), consistent with previous studies on primary cortical progenitors (Cheng et al., 2006; Hu et al., 2008), *in vivo* gene expression (e.g. Furuta et al., 1997; Currle et al., 2005), and roof plate or BMP receptor ablation phenotypes (Cheng et al., 2006; Hébert et al., 2002). Thus, high BMP consistently suppresses cortical gene expression and cortical fate.

Perhaps more interestingly, intermediate BMP4 concentrations (i.e. the dorsomedial cortex regime) upregulated DV-graded *Emx2* and *Lhx2*, but not VD-graded genes *Pax6* or *Foxg1* (Fig. 3), which is concordant with the *in vivo* phenotypes of these four genes following roof plate ablation (Cheng et al., 2006). The concordant findings for *Emx2* and *Lhx2* are also consistent with the BMP-responsive enhancer in the *Emx2* gene (Theil et al., 1999) and with *Lhx2* upregulation at a distance from BMP4-soaked beads in explants (Monuki et al., 2001). However, Ngn2 findings are discordant; *Ngn2* was upregulated by intermediate BMP4 here (Fig. 3), but unaffected by roof plate ablation (Cheng et al., 2006). Collectively, these findings support a model in which intermediate BMP signaling upregulates *Emx2* and *Lhx2* in dorsomedial cortex/hippocampal anlagen, thereby generating their DV gradients, while VD genes must rely on signals other than BMPs to sculpt their gradients (Cheng et al., 2006). (Note: the BMP4-mediated Ngn2 upregulation seen here would create an Ngn2 gradient of the wrong polarity, i.e. it would have DV rather than the VD polarity seen normally.)

### *Lhx2* independence of BMP morphogenic activity

*Lhx2* inactivation had no demonstrable effect on any aspect of BMP4-mediated CH and CPEC fate acquisition (levels, dose-response profiles, or kinetics). Thus, *Lhx2* neither mediates nor regulates the DTM morphogenic activities of BMP4. While the *in vitro* data are clear, they are perplexing given the DTM-related phenotypes of *Lhx2* null mice. Constitutive *Lhx2* null mice have excess CH and CPECs (Monuki et al., 2001), and mosaic *Lhx2* inactivation in dorsomedial cortex leads to ectopic CH (Mangale et al., 2008), indicating that *Lhx2* genetically suppresses DTM fates, but does not do so by regulating BMP signaling intensity (Doan et al., 2012).

As such, an explanation for the different *Lhx2* null phenotypes will require further exploration. Spatial and temporal differences in *Lhx2* inactivation could account for the differences, since early *in vitro* inactivations (more analogous to the constitutive *Lhx2* state) were not possible here due to poor neuroepithelial induction, which is not apparent *in vivo* (Mangale et al., 2008; Monuki et al., 2001). This alone suggests important qualitative differences between the *in vitro* and *in vivo* systems. Positional determinants lost in culture could also be responsible, since *Lhx2* null fates *in vivo* critically depend on position within the dorsal telencephalon (Mangale et al., 2008).

### Lhx2 selector activity and BMP-mediated cortical patterning

*In vivo*, *Lhx2* acts at or near the top of the genetic hierarchy for selecting cortical identity (Mangale et al., 2008), and our *in vitro* findings here are consistent with this concept. At intermediate BMP4 concentrations, *Lhx2* upregulation (at 7 DIV) preceded the upregulations of other cortical genes (*Emx2* and *Ngn2* at 10 DIV) in both mESC lines (Fig. 3), and all cortical markers were reduced upon *Lhx2* inactivation (Fig. 5). Abrogation of BMP4-mediated *Pax6* suppression due to *Lhx2* loss (Fig. 5) is also consistent with previous *Lhx2*-*Pax6* epistasis studies (Hou et al., 2013; Mangale et al., 2008; Porter et al., 1997; Tetreault et al., 2009). Interestingly, published examples indicate that *Lhx2* can regulate *Pax6* either positively or negatively, highlighting the importance of *Lhx2*-*Pax6* context. Other than *Pax6*, however, *Lhx2* presence or absence had no apparent effect on BMP4-mediated regulation of the other cortical genes. As with DTM fates, this implies that BMP morphogenic activity in the dorsal telencephalon is largely *Lhx2*-independent; however, BMP consistently regulates *Lhx2* expression (suppression at high concentrations, upregulation at intermediate ones), suggesting a BMP-Lhx2 pathway with little to no feedback.

## MATERIALS AND METHODS

### Mice

Mouse colonies and breeding were performed under Institutional Animal Care and Use Committee guidelines. Noon of the vaginal plug date was designated as day 0.5 for timed pregnancies. Crown-rump length was measured to verify embryonic ages. Wild-type mice (CD1) were obtained from Charles River Laboratories (Wilmington, MA) and *Emx1^Cre/+^Lhx2^cKO/sKO^* were derived as described (Mangale et al., 2008).

### Mouse ESC line derivation and expansion

The lines M1 (*R26^CreER/+^Lhx2^cKO/cKO^*, C57BL/6J with minor CD1 background), M2 (*Ttr::RFP* hemizygous, mostly CD1 background with C57BL/6J and ICR), and M4 (*R26^CreER/+^*, C57BL/6J with minor CD1 background) were derived, cultured and verified for pluripotency, chromosomal numbers and mycoplasma negativity as described (Watanabe et al., 2012). The M2 line was derived by the UCI Transgenic Mouse Facility using the 2i method (Li et al., 2008). Prior to all experiments, frozen mESCs were cultured for at least two passages after thawing. Experiments were conducted on cells between passage numbers 11 and 40.

### Mouse ESC culture and differentiation

Neural differentiations in ‘SFEBq’ aggregates were performed as described (Eiraku et al., 2008; Watanabe et al., 2012). For the ‘aggregate’ method, cells were maintained as aggregates for the entire culture period. After 5 DIV, differentiation media (Eiraku et al., 2008) was replaced with fresh media containing 0.15-150 ng/ml BMP4 (R&D Systems, Minneapolis, MN, USA) for another 5 days (10 days total). For the ‘monolayer’ method, 5-day aggregates were dissociated to single cells using TrypLE Express (Life Technologies, Carlsbad, CA, USA) and plated onto PDL/laminin-coated plates at 2.5-5×10^5^ (M1) or 7-10×10^5^ (M2) cells/cm^2^ with BMP4 in fresh differentiation media for 5 days. Thus, for both the aggregate and monolayer methods, total culture time was 10 days, and BMP4 exposure duration was 5 days. BMP4 was applied only once in each experiment.

### Primary cortical progenitor culture

For *Emx1^Cre/+^Lhx2^cKO/sKO^* studies (Mangale et al., 2008), E11.5 cortical progenitor cells were dissected and cultured as described (Hu et al., 2008), except that BMP4 was added during cell resuspension prior to plating.

### RT-qPCR and immunostaining

RT-qPCR was performed as described (Currle et al., 2005; Hu et al., 2008) on LightCycler^®^ 480 System (Roche, Indianapolis, IN, USA) using 18S normalization, Microsoft Excel for statistical tests (two-tailed *t*-tests assuming equal variance, asterisks for *P* values <0.05), and KaleidaGraph (Synergy Software) for graphing. All primers and amplicons were validated as described (Currle et al., 2005; Hu et al., 2008). Primer sequences are listed in Table S1. All RT-qPCR studies are reported as means and standard errors (s.e.m.) for at least two biological replicates representing cultures initiated on different days. Immunostaining was performed as described (Cheng et al., 2006; Currle et al., 2005; Hu et al., 2008; Mangale et al., 2008; Watanabe et al., 2012). Antibodies and counterstains used: Lmx1a (Millipore AB10533, 1:2000, Billerica, MA, USA); Msx1/2 (DHSB 4G1, 1:100, Iowa City, IA, USA); Alexa 488-, 555-, and 633-conjugated secondary antibodies (Molecular Probes, Grand Island, NY, USA, 1:100); Hoechst 33342 (Molecular Probes).

### Western blot

Protein extraction was performed using RIPA lysis buffer supplemented with protease inhibitor and phosphatase inhibitor cocktails (Roche). Cell lysates were collected and incubated on ice for 10 min. Lysates were centrifuged at 10,000 ***g*** for 15 min, and supernatant was transferred and stored at −80˚C. Laemmli sample buffer (Bio-Rad, Hercules, CA, USA) was added to samples and ran on 7.5% Mini-Protean TGX gel (Bio-Rad) in Tris Glycine SDS buffer (Bio-Rad). Samples were transferred onto 0.45 μm nitrocellulose membranes (Bio-Rad) in Tris Glycine transfer buffer overnight. Primary antibodies used: beta-actin (Cell Signaling, Danvers, MA, USA; 3700), SMAD1 (Santa Cruz Biotechnology sc7965, Santa Cruz, CA, USA), pSMAD1/5/8 (Millipore AB3848). Secondary antibodies used: IR Dye 680LT anti-rabbit IgG (LI-COR Biosciences, Lincoln, NE, USA), IR Dye 800CW anti-mouse IgG (LI-COR). Membranes were scanned and quantified using Odyssey IR scanner (LI-COR Biosciences). Studies are reported as means and standard errors for at least three biological replicates representing cultures initiated on different days.

### Imaging

Epifluorescence and brightfield imaging and processing were performed as described (Hu et al., 2008; Mangale et al., 2008; Watanabe et al., 2012). Confocal images and Z-stacks were acquired using a Zeiss LSM510 confocal microscope. Epifluorescence imaging was done on a Nikon Eclipse Ti and captured using Nikon NIS-Elements AR3.00 software. Epifluorescence imaging on live cell aggregates was done on an EVOS^®^ fl Digital Fluorescence Microscope (Life Technologies). All images were compiled in Adobe Photoshop, with image adjustments restricted to brightness, contrast, and levels to the entire field. Any images used for comparisons were acquired and processed in parallel using identical settings and adjustments for presentation and quantification.

### Cell quantification

Lmx1a, Msx1/2, Ttr::RFP, and Hoechst were manually counted in Photoshop or ImageJ from two biological replicates and four different confocal planes in a blinded manner. Denominators included all Hoechst-stained cells. For blinded scoring of immunostains, matched unprocessed confocal images (Zeiss LSM 510) were scored. 1000-2000 cells were counted for each condition. Excel was used for statistical tests (two-tailed *t*-tests assuming equal variance, asterisks for *P* values <0.05).

## References

[BIO012021C1] Ashe H. L. and Briscoe J. (2006). The interpretation of morphogen gradients. *Development* 133, 385-394. 10.1242/dev.02238 16410409

[BIO012021C2] Cheng X., Hsu C.-m., Currle D. S., Hu J. S., Barkovich A. J. and Monuki E. S. (2006). Central roles of the roof plate in telencephalic development and holoprosencephaly. *J. Neurosci.* 26, 7640-7649. 10.1523/JNEUROSCI.0714-06.2006 16855091 PMC6674267

[BIO012021C3] Chizhikov V. V., Lindgren A. G., Mishima Y., Roberts R. W., Aldinger K. A., Miesegaes G. R., Currle D. S., Monuki E. S. and Millen K. J. (2010). Lmx1a regulates fates and location of cells originating from the cerebellar rhombic lip and telencephalic cortical hem. *Proc. Natl. Acad. Sci. USA* 107, 10725-10730. 10.1073/pnas.0910786107 20498066 PMC2890798

[BIO012021C4] Cornell R. A. and Ohlen T. V. (2000). Vnd/nkx, ind/gsh, and msh/msx: conserved regulators of dorsoventral neural patterning? *Curr. Opin. Neurobiol.* 10, 63-71. 10.1016/S0959-4388(99)00049-5 10679430

[BIO012021C5] Currle D. S., Cheng X., Hsu C.-m. and Monuki E. S. (2005). Direct and indirect roles of CNS dorsal midline cells in choroid plexus epithelia formation. *Development* 132, 3549-3559. 10.1242/dev.01915 15975937

[BIO012021C6] Dessaud E., Yang L. L., Hill K., Cox B., Ulloa F., Mynett A., Novitch B. G. and Briscoe J. (2007). Interpretation of the sonic hedgehog morphogen gradient by a temporal adaptation mechanism. *Nature* 450, 717-720. 10.1038/nature06347 18046410

[BIO012021C7] Doan L. T., Javier A. L., Furr N. M., Nguyen K. L., Cho K. W. and Monuki E. S. (2012). A Bmp reporter with ultrasensitive characteristics reveals that high Bmp signaling is not required for cortical hem fate. *PLoS ONE* 7, e44009 10.1371/journal.pone.0044009 22984456 PMC3439469

[BIO012021C8] Eiraku M., Watanabe K., Matsuo-Takasaki M., Kawada M., Yonemura S., Matsumura M., Wataya T., Nishiyama A., Muguruma K. and Sasai Y. (2008). Self-organized formation of polarized cortical tissues from ESCs and its active manipulation by extrinsic signals. *Cell Stem Cell* 3, 519-532. 10.1016/j.stem.2008.09.002 18983967

[BIO012021C9] Ericson J., Rashbass P., Schedl A., Brenner-Morton S., Kawakami A., van Heyningen V., Jessell T. M. and Briscoe J. (1997). Pax6 controls progenitor cell identity and neuronal fate in response to graded Shh signaling. *Cell* 90, 169-180. 10.1016/S0092-8674(00)80323-2 9230312

[BIO012021C10] Fernandes M., Gutin G., Alcorn H., McConnell S. K. and Hébert J. M. (2007). Mutations in the BMP pathway in mice support the existence of two molecular classes of holoprosencephaly. *Development* 134, 3789-3794. 10.1242/dev.004325 17913790

[BIO012021C11] Furuta Y., Piston D. W. and Hogan B. (1997). Bone morphogenetic proteins (BMPs) as regulators of dorsal forebrain development. *Development* 124, 2203-2212. 9187146 10.1242/dev.124.11.2203

[BIO012021C12] Grove E. A. and Monuki E. S. (2013). Morphogens, patterning centers, and their mechanisms of action. In *Comprehensive Developmental Neuroscience: Patterning and Cell Type Specification in the Developing CNS and PNS* (ed. Rakic P. and Rubenstein J.), pp. 26-41. Oxford, UK: Academic Press.

[BIO012021C13] Hébert J. M., Mishina Y. and McConnell S. K. (2002). BMP signaling is required locally to pattern the dorsal telencephalic midline. *Neuron* 35, 1029-1041. 10.1016/S0896-6273(02)00900-5 12354394

[BIO012021C14] Hou P.-S., Chuang C.-Y., Kao C.-F., Chou S.-J., Stone L., Ho H.-N., Chien C.-L. and Kuo H.-C. (2013). LHX2 regulates the neural differentiation of human embryonic stem cells via transcriptional modulation of PAX6 and CER1. *Nucleic Acids Res.* 16, 7753-7770. 10.1093/nar/gkt567 PMC3763550 23804753

[BIO012021C15] Hu J. S., Doan L. T., Currle D. S., Paff M., Rheem J. Y., Schreyer R., Robert B. and Monuki E. S. (2008). Border formation in a Bmp gradient reduced to single dissociated cells. *Proc. Natl. Acad. Sci. USA* 105, 3398-3403. 10.1073/pnas.0709100105 18292231 PMC2265170

[BIO012021C16] Kicheva A., Pantazis P., Bollenbach T., Kalaidzidis Y., Bittig T., Jullicher F. and Gonzalez-Gaitan M. (2007). Kinetics of morphogen gradient formation. *Science* 315, 521-525. 10.1126/science.1135774 17255514

[BIO012021C17] Kicheva A. and González-Gaitán M. (2008). The decapentaplegic morphogen gradient: a precise definition. *Curr. Opin. Cell Biol.* 20, 137-143. 10.1016/j.ceb.2008.01.008 18329870

[BIO012021C18] Li P., Tong C., Mehrian-Shai R., Jia L., Wu N., Yan Y., Maxson R. E., Schulze E. N., Song H., Hsieh C.-L. et al. (2008). Germline competent embryonic stem cells derived from rat blastocysts. *Cell* 135, 1299-1310. 10.1016/j.cell.2008.12.006 19109898 PMC2735113

[BIO012021C19] Louvi A., Yoshida M. and Grove E. A. (2007). The derivatives of the Wnt3a lineage in the nervous system. *J. Compara. Neurol.* 504, 550-569. 10.1002/cne.21461 17701978

[BIO012021C20] Mabie P. C., Mehler M. F. and Kessler J. A. (1999). Multiple roles of bone morphogenetic protein signaling in the regulation of cortical cell number and phenotype. *J. Neurosci.* 19, 7077-7088. 10436062 10.1523/JNEUROSCI.19-16-07077.1999 PMC6782885

[BIO012021C21] Mangale V. S., Hirokawa K. E., Satyaki P. R. V., Gokulchandran N., Chikbire S., Subramanian L., Shetty A. S., Martynoga B., Paul J., Mai M. V. et al. (2008). Lhx2 selector activity specifies cortical identity and suppresses hippocampal organizer fate. *Science* 319, 304-309. 10.1126/science.1151695 18202285 PMC2494603

[BIO012021C22] Massagué J., Seoane J. and Wotton D. (2005). Smad transcription factors. *Genes Dev.* 19, 2783-2810. 10.1101/gad.1350705 16322555

[BIO012021C23] Meyer G. (2010). Building a human cortex: the evolutionary differentiation of Cajal-Retzius cells and the cortical hem. *J. Anat.* 217, 334-343. 10.1111/j.1469-7580.2010.01266.x 20626498 PMC2992412

[BIO012021C24] Meyer G., Perez-Garcia C. G., Abraham H. and Caput D. (2002). Expression of p73 and Reelin in the developing human cortex. *J. Neurosci.* 22, 4973-4986. 12077194 10.1523/JNEUROSCI.22-12-04973.2002 PMC6757727

[BIO012021C25] Molyneaux B. J., Ariotta P., Menezes J. R. L. and Macklis J. D. (2007). Neuronal subtype specification in the cerebral cortex. *Nat. Neurosci. Rev.* 8, 427-437. 10.1038/nrn2151 17514196

[BIO012021C26] Monuki E. S., Porter F. D. and Walsh C. A. (2001). Patterning of the dorsal telencephalon and cerebral cortex by a roof plate-Lhx2 pathway. *Neuron* 32, 591-604. 10.1016/S0896-6273(01)00504-9 11719201

[BIO012021C27] Porcher A. and Dostatni N. (2010). The bicoid morphogen system. *Curr. Biol.* 20, R249-R254. 10.1016/j.cub.2010.01.026 20219179

[BIO012021C28] Porter F. D., Drago J., Xu Y., Cheema S. S., Wassif C., Huang S. P., Lee E., Gringerg A., Massalas J. S., Bodine D. et al. (1997). Lhx2, a LIM homeobox gene, is required for eye, forebrain, and definitive erythrocyte development. *Development* 124, 2935-2944. 10.1242/dev.124.15.2935 9247336

[BIO012021C29] Ramos C. and Robert B. (2005). msh/Msx gene family in neural development. *Trends. Genet.* 21, 624-632. 10.1016/j.tig.2005.09.001 16169630

[BIO012021C30] Ramos C., Martinez A., Robert B. and Soriano E. (2004). Msx1 expression in the adult mouse brain: characterization of populations of beta-galactosidase-positive cells in the hippocampus and fimbria. *Neuroscience* 127, 893-900. 10.1016/j.neuroscience.2004.06.010 15312901

[BIO012021C31] Schilling T. F., Nie Q. and Lander A. D. (2012). Dynamics and precision in retinoic acid morphogen gradients. *Curr. Opin. Genet. Dev.* 22, 562-569. 10.1016/j.gde.2012.11.012 23266215 PMC3790664

[BIO012021C32] Srinivasan S., Hu J. S., Currle D. S., Fung E. S., Hayes W. B., Lander A. D. and Monuki E. S. (2014). A BMP-FGF morphogen toggle switch drives the ultrasensitive expression of multiple genes in the developing forebrain. *PLoS Comput. Biol.* 10, e1003463 10.1371/journal.pcbi.1003463 24550718 PMC3923663

[BIO012021C33] Stamataki D., Ulloa F., Tsoni S. V., Mynett A. and Briscoe J. (2005). A gradient of Gli activity mediates graded Sonic Hedgehog signaling in the neural tube. *Genes Dev.* 19, 626-641. 10.1101/gad.325905 15741323 PMC551582

[BIO012021C34] Tabata T. and Takei Y. (2004). Morphogens, their identification and regulation. *Development* 131, 703-712. 10.1242/dev.01043 14757636

[BIO012021C35] Tetreault N., Champagne M.-P. and Bernier G. (2009). The LIM homeobox transcription factor Lhx2 is required to specify the retina field and synergistically cooperates with Pax6 for Six6 trans-activation. *Dev. Biol.* 327, 541-550. 10.1016/j.ydbio.2008.12.022 19146846

[BIO012021C36] Theil T., Alvarez-Bolado G., Walter A. and Rüther U. (1999). Gli3 is required for Emx gene epression during dorsal telencephalon development. *Development* 126, 3561-3571. 10409502 10.1242/dev.126.16.3561

[BIO012021C37] Thomas T. and Dziadek M. (1993). Capacity to form choroid plexus-like cells in vitro is restricted to specific regions of the mouse neural ectoderm. *Development* 117, 253-262. 8223250 10.1242/dev.117.1.253

[BIO012021C38] Tole S. and Grove E. A. (2001). Detailed field pattern is intrinsic to the embryonic mouse hippocampus early in neurogenesis. *J. Neurosci.* 21, 1580-1589. 11222648 10.1523/JNEUROSCI.21-05-01580.2001 PMC6762964

[BIO012021C39] Toyoda R., Assimacopoulos S., Wilcoxon J., Taylor A., Feldman P., Suzuki Hirano A., Shimogori T. and Grove E. A. (2010). FGF8 acts as a classic diffusible morphogen to pattern the neocortes. *Development* 137, 3439-3448. 10.1242/dev.055392 20843859 PMC2947756

[BIO012021C40] Tozer S., Le Dreau G., Marti E. and Briscoe J. (2013). Temporal control of BMP signalling determines neuronal subtype identity in the dorsal neural tube. *Development* 140, 1467-1474. 10.1242/dev.090118 23462473 PMC3596990

[BIO012021C41] Watanabe M., Kang Y.-J., Davies L. M., Meghpara S., Lau K., Chung C.-Y., Kathiriya J., Hadjantonakis A.-K. and Monuki E. S. (2012). BMP4 sufficiency to induce choroid plexus epithelial fate from embryonic stem cell-derived neuroepithelial progenitors. *J. Neurosci.* 32, 15934-15945. 10.1523/JNEUROSCI.3227-12.2012 23136431 PMC3505486

[BIO012021C42] Zou H.-L., Su C.-J., Shi M., Zhao G.-Y., Li Z.-Y., Guo C. and Ding Y.-Q. (2009). Expression of the LIM-homeodomain gene Lmx1a in the postnatal mouse central nervous system. *Brain Res. Bull.* 78, 306-312. 10.1016/j.brainresbull.2008.12.001 19111912

